# The Scaling Limit of Random Two-Connected Series–Parallel Maps

**DOI:** 10.1007/s10959-026-01537-x

**Published:** 2026-08-27

**Authors:** Daniel Amankwah, Jakob Björnberg, Sigurdur Örn Stefánsson, Benedikt Stufler, Joonas Turunen

**Affiliations:** 1 Mathematics division, University of Iceland, Reykjavik, Iceland; 2 Department of Mathematics, Chalmers University of Technology, Gothenburg, Sweden; 3 Department of Mathematics, University of Gothenburg, Gothenburg, Sweden; 4 Institute of Discrete Mathematics and Geometry, TU Wien, Vienna, Austria; 5 University of Helsinki, Helsinki, Finland

**Keywords:** Random planar map, Simply generated trees, Scaling limits, Brownian tree, 05C80, 60F17, 60J80

## Abstract

A finite graph embedded in the plane is called a series–parallel map if it can be obtained from a finite tree by repeatedly subdividing and doubling edges. We study the scaling limit of weighted random two-connected series–parallel maps with *n* edges and show that under fairly general integrability conditions on these weights, the maps with distances rescaled by a factor $n^{-1/2}$ converge to a constant multiple of Aldous’ continuum random tree (CRT) in the Gromov–Hausdorff sense. The proof relies on a bijection between a set of trees with *n* leaves and a set of series–parallel maps with *n* edges, together with a novel *blob decomposition* of the maps.

## Introduction

A finite connected graph is called a *series–parallel graph* if it can be obtained from a finite tree by repeatedly subdividing or doubling edges. It may equivalently be characterized as a graph that does not contain the complete graph $K_{4}$ as a minor. In this work, we consider series–parallel *maps*, which are series–parallel graphs embedded in the plane, viewed up to continuous deformations.

**Fig. 1:**
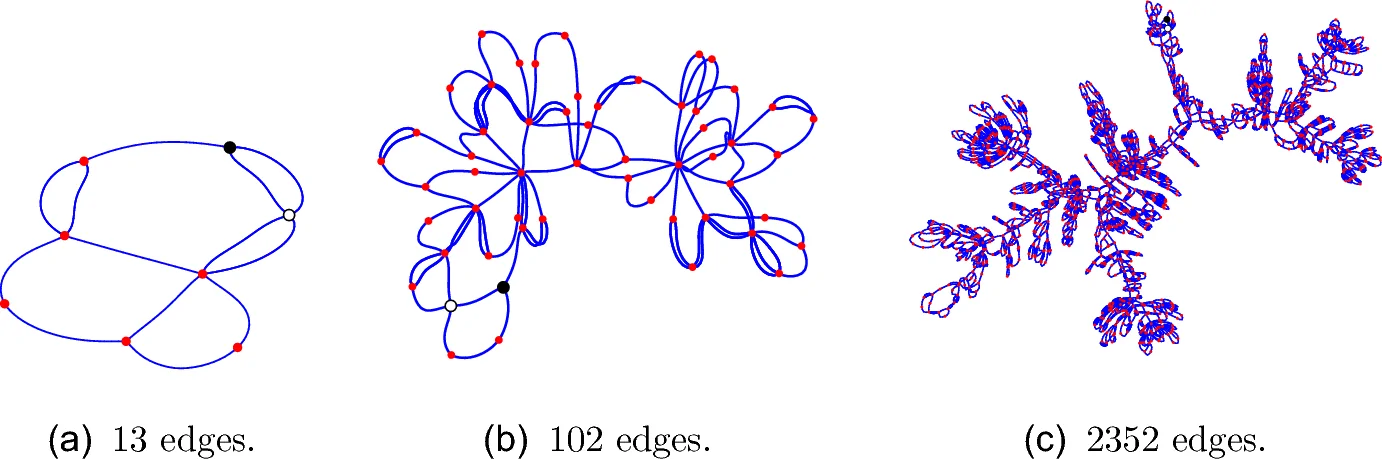
A simulation of uniform two-connected series–parallel maps with different numbers of edges. The endpoints of the root edge are represented by ∙ and ∘

Specifically, we consider series–parallel maps that are *two-connected*, and we are interested in their *scaling limits*. Without the condition of two-connectivity, uniformly sampled series–parallel maps have Aldous’ continuum random tree (CRT) as their scaling limit, as easily follows from a general theorem of Stufler [21, Thm. 6.62]. More precisely, uniform samples of such maps with *n* edges, and distances rescaled by $n^{-1/2}$, converge weakly in the Gromov–Hausdorff sense to a multiple of the CRT. The essence of the proof lies in decomposing the map into two-connected components, or *blocks*, and using a coupling with simply generated trees where each block of the map corresponds to a vertex in the tree. As the tree scales at the order $n^{1/2}$, and since blocks are typically small, after rescaling distances by $n^{-1/2}$ on average each block contributes a constant length to the geodesic. Thus, the global shape of the rescaled map is approximated by the tree, stretched by a constant factor.

In this work, however, we would like to understand the structure of the blocks themselves. Thus, we focus our attention on *two-connected* series–parallel maps. These do not enjoy the same type of coupling to simply generated trees and prove more difficult to handle. Our main result is that, nevertheless, uniformly sampled two-connected series–parallel maps with *n* edges converge, as $n\rightarrow \infty$ with distances rescaled by $n^{-1/2}$, to a multiple of the CRT. Figure 1 shows simulations of these uniformly sampled maps and suggests that they become tree-like with an increasing number of edges.

Analogous results have been established before for several tree-like random maps, which can often be characterized through excluded minors. Most fundamental is the original result of Aldous, showing that uniform trees with *n* edges converge to the CRT when rescaled by $n^{-1/2}$ [1–3]. Trees may be characterized by not containing $K_3$ as a minor. Other notable cases are various classes of outerplanar graphs. A graph is called *outerplanar* if it may be embedded in the plane so that every vertex lies on the boundary of the external face; equivalently, they are graphs not containing $K_4$ or the complete bipartite graph $K_{3,2}$ as minors. In [8], Caraceni established that uniformly sampled outerplanar maps (properly rescaled) converge to the CRT. Stufler [21] generalized this result to a family of face-weighted outerplanar maps, using the aforementioned coupling with simply generated trees. Note that outerplanar maps are series–parallel maps, since series–parallel maps have $K_4$ as an excluded minor, and outerplanar maps further exclude $K_{3,2}$ as a minor.

If one additionally imposes the condition of two-connectivity on outerplanar maps, one obtains *dissections of a polygon*. Random, face-weighted dissections of polygons were studied by Curien, Haas, and Kortchemski [9] who showed, under a suitable condition on the weights, that the scaling limit is a constant multiple of the CRT. Their proof involves relating the dissections bijectively to trees and using a clever Markov chain argument to compare lengths of geodesics in the dissections and their corresponding trees.

One of the main aims of the above studies is to understand how universally the CRT appears as a scaling limit for different models of random graphs or maps. Note that planar maps themselves, given by excluding $K_5$ and $K_{3,3}$ as minors, behave vastly differently. Uniform planar maps with *n* edges, when rescaled by $n^{-1/4}$, admit the so-called *Brownian sphere* as a scaling limit [4], which is completely different from the CRT (see Table 1 for a summary of these results).

**Table 1:** Scaling limits of uniform maps with *n* edges characterized by exclusion of minors

Map	Excluded minors	Diameter	Scaling limit
Plane trees	$K_3$	$n^{1/2}$	CRT
Outerplanar maps	$K_{4}$, $K_{2,3}$	$n^{1/2}$	CRT
Series–parallel maps	$K_4$	$n^{1/2}$	CRT
Planar maps	$K_5, K_{3,3}$	$n^{1/4}$	Brownian sphere

Another motivation for understanding the geometry of series–parallel maps better stems from their conjectured connection to the two-dimensional Liouville quantum gravity (LQG), as a part of a more general framework of random surfaces obtained as a mating of trees (see [11]). The exact connection could be figured out via random separable permutations, which is outlined in Sect. 4 at the end of this work. The geometric interpretation of SP maps in the context of LQG has been under active discussion in the random geometry research community, but to our knowledge, nothing has been proven so far. One of the very few ideas is the conjecture that conformally embedded rooted rescaled series–parallel maps converge toward the 2-LQG quantum sphere under a suitable conformal embedding [6].

We use a bijection with certain plane trees (see Sect. 2.3 for details), which enables us to describe the distribution of the random maps with *n* edges in terms of a Bienaymé–Galton–Watson (BGW) tree conditioned on having *n* leaves. In order to study the geodesics in the map, we introduce a novel blob decomposition of series–parallel maps. Roughly speaking, such maps get glued together from smaller maps, with the gluing operation identifying a pair of vertices in one blob with a pair of vertices in another blob. This way, the resulting pair of vertices forms a two-separator of the map. Any geodesics joining vertices from opposite sides are then forced to pass through one of the two gluing points. This paves the way for applying concentration inequalities for Markov-additive processes in order to handle the geodesics. However, the stationary distribution is non-explicit and almost intractable. In order to tackle this, we use a combinatorial trick to carefully define our blobs in a way so that any pair of gluing points is adjacent in at least one of the two blobs. This trick reduces the state space of our Markov-additive process from being infinite to having a finite number of states, thus crucially reducing the complexity for verifying necessary conditions for concentration inequalities.

Moreover, the relation with BGW trees allows us to consider a weighted version of the random maps in a natural way by assigning a general offspring distribution μ to the BGW trees. We will assume that this offspring distribution is critical and that it has finite exponential moments (is light-tailed), which includes the uniform distribution as a special case. The precise definition of the weighted series–parallel maps is given in Definition 2.2 in Sect. 2.6.

The CRT $\mathcal {T}_\textbf{e}$ used in this work is the version constructed from a normalized Brownian excursion $\textbf{e}$ (see [16]). Let $\textsf{SP}^\mu _n$ denote the random, weighted, rooted, two-connected series–parallel map with *n* non-root edges corresponding to a critical offspring distribution μ of a BGW process possessing finite exponential moments (see Definition 2.2). The following is our main result:

### Theorem 1.1

Let $d_{\textsf{SP}^\mu _n}$ denote the usual graph metric on $\textsf{SP}^\mu _n$. There exists a constant $c_{\mu }>0$ such that

$$ (\textsf{SP}^\mu _n, c_{\mu } n^{-1/2} d_{\textsf{SP}^\mu _n}) \,{\buildrel d \over \longrightarrow }\,(\mathcal {T}_{\textrm{e}}, d_{\mathcal {T}_{\textrm{e}}}) $$

in the Gromov–Hausdorff sense.

The scaling constant $c_\mu$ remains implicit because it is defined in terms of a Markov-additive process with implicitly defined transition probabilities (see Remark 3.4).

We also obtain optimal tail bounds for the diameter $\textrm{D}(\textsf{SP}^\mu _n)$:

### Theorem 1.2

There exist constants C,c>0 such that for all *n* and all x>0

$$ \mathbb {P}(\textrm{D}(\textsf{SP}^\mu _n) > x) \le C \exp (-c x^2 /n). $$

### Remark 1.3

We will consider other closely related classes of series–parallel maps referred to as series–parallel networks. Their definitions are given in Subsection 2.1. The two previous theorems also apply to random weighted series–parallel networks with the same scaling constant $c_\mu$.

## Decomposing and Sampling Series–Parallel Maps

### Rooted, Two-Connected Series–Parallel Maps

A planar map is a drawing of a finite connected graph in the plane so that edges do not cross except possibly at their endpoints, viewed up to orientation preserving homeomorphisms of the plane. A series–parallel (SP) map is a planar map, which does not contain the complete graph $K_4$ as a minor. The objects of interest in this paper are two-connected SP maps. Another way to describe them is to start from a double edge and repeatedly subdivide or double edges [10, Theorems 1 and 3].

**Fig. 2:**
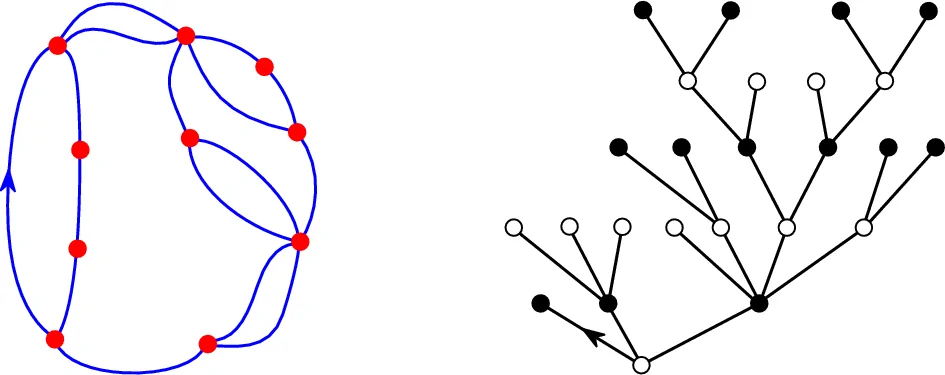
Left: An example of a rooted, two-connected SP map. The directed root edge is indicated by an arrow. Right: Its corresponding labeled plane tree where ∘ denotes the label *P* and ∙ denotes the label *S*

We will assign a direction to one of the edges of the map and refer to it as the *root* of the map. The root will be denoted by $e=(*_0,*_1)$. For concreteness, we draw the maps so that the root edge lies on the boundary of the unbounded face and is directed in a clockwise manner along that face (see Fig. 2 (left) for an example).

Denote the set of rooted two-connected SP maps with n≥1 non-root edges by $\mathcal{S}\mathcal{P}_{n}$ and the set of all such finite maps by $\mathcal{S}\mathcal{P}=\cup _{n\ge 1} \mathcal{S}\mathcal{P}_n$. We deliberately leave out the map with the single root edge for notational convenience.

In order to be able to describe a decomposition of the maps into series and parallel components, we require two additional definitions. For $$M \in \mathcal{S}\mathcal{P}_{n}$$, let $M^{\prime }$ be the map obtained by removing the root edge from *M*, keeping the endpoints $$*_0,*_1$$ as marked vertices of $M^{\prime }$. Define for all n≥2

$$\begin{aligned} \mathcal {S}_n&= \{M' ~|~ M \in \mathcal{S}\mathcal{P}_{n}, M' \text { is not two-connected}\}, \\ \mathcal {P}_n&= \{M' ~|~ M \in \mathcal{S}\mathcal{P}_{n}, M' \text { is two-connected} \}. \end{aligned}$$

Also set

2.1 $$\begin{aligned} \mathcal {S}= \cup _{n\ge 2} \mathcal {S}_n, \qquad \mathcal {P}= \cup _{n\ge 2}\mathcal {P}_n,\qquad \text {and} \qquad \mathcal {N}= \mathcal {S}\cup \mathcal {P}\cup \{e\}. \end{aligned}$$

The operation of removing or adding back the root edge defines a bijection

**Fig. 3:**
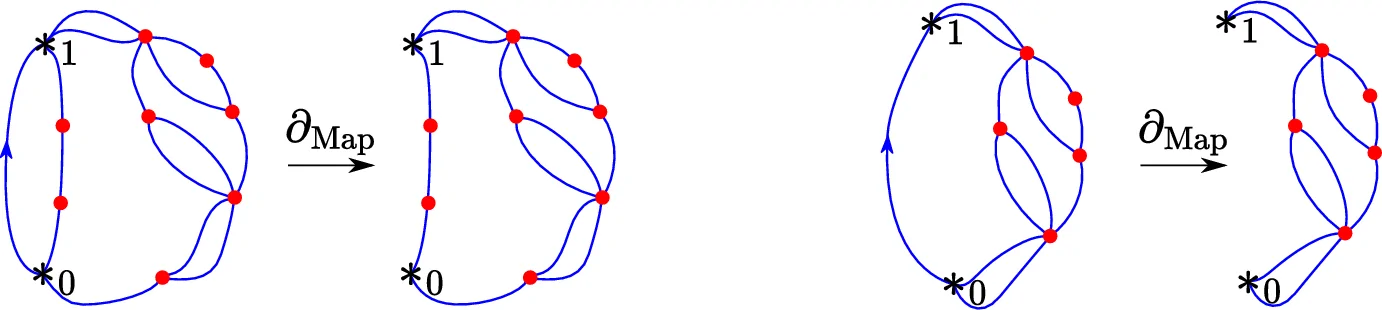
The root edge is removed from a rooted two-connected SP map. The resulting map is either two-connected (left) or it is not (right)

2.2 $$\begin{aligned} \partial _\textrm{Map}: \mathcal{S}\mathcal{P}\rightarrow \mathcal {N}, \end{aligned}$$

see Fig. 3. We refer to the maps in $\mathcal {N}$ as *series–parallel (SP) networks*.

### Plane Trees and Labels

Recall the standard definition of the Ulam–Harris tree: set $\mathcal {U}=\bigcup _{n=0}^{\infty } \mathbb {N}^{n},$ where $\mathbb {N}=\{1,2,3,\dotsc \}$ and $\mathbb {N}^{0}=\{\varnothing \}$ by convention. If $u=u^{1},\dots ,u^{m}$ and $v=v^{1},\dots ,v^{n}$ are elements of $\mathcal {U}$, we write the concatenation of *u* and *v* as $uv=u^{1},\dots ,u^{m},v^{1},\dots ,v^{n}$.

#### Definition 2.1

A *plane tree* *T* is a finite or infinite subset of $\mathcal {U}$ such that

1. $$\varnothing \in T$$,
2. if v∈T and v=uj for some $j \in \mathbb {N}$, then u∈T.
3. for every u∈T, there exists an integer $\operatorname {out}_T(u) \ge 0$ (the outdegree or number of children of *u*) such that for every $j \in \mathbb {N}$, uj∈T if and only if $1 \le j \le \operatorname {out}_T(u)$.

The element $\varnothing \in \mathcal {U}$ is called the *root vertex*, and the pair $\{\varnothing ,1\}$ is called the *root edge*. One may view plane trees as planar maps with a directed root edge, which do not contain a cycle. A vertex of outdegree 0 is called a *leaf*, and an edge incident to a leaf is called a *leaf edge*. The trivial tree consisting only of the root vertex ∅, which is then a leaf, will be denoted by ∙.

We denote by $\mathcal {T}$ the set of *finite* plane trees, which have no vertices of outdegree 1, and by $\mathcal {T}_n\subseteq \mathcal {T}$ the set of such trees with n≥1 leaves. We will apply labels *S* and *P* to the trees in $\mathcal {T}$ (related to the series and parallel decompositions of maps). We use the notation $\bar{S} = P$ and $\bar{P} = S$ when we need to swap labels. If $(T,\ell )\in \mathcal {T}\times \{S,P\}$ and $T\ne \bullet$, we say that *T* is labeled by ℓ and we assign the label ℓ to vertices in every even generation and $\bar{\ell }$ to vertices in every odd generation. In the case, when T=∙, we take (∙,P) and (∙,S) to be equivalent and simply denote them by ∙ and say that ∙ is neither labeled by *P* nor *S*. Define $\mathcal {T}\mathcal {P}= (\mathcal {T}{\setminus }\{\bullet \})\times \{P\}$ and $\mathcal {T}\mathcal {S}= (\mathcal {T}{\setminus }\{\bullet \})\times \{S\}$ as the sets of nontrivial trees labeled by *P* and *S*, respectively, and let

2.3 $$\begin{aligned} \mathcal {T}\mathcal {N}:= \mathcal {T}\mathcal {P}\cup \mathcal {T}\mathcal {S}\cup \{\bullet \}. \end{aligned}$$

The corresponding sets of trees with *n* leaves are denoted using the lower index *n*.

Next define $\mathcal {T}^*\subseteq \mathcal {T}$ as the set of trees in $\mathcal {T}$ such that the leftmost child of the root is a leaf, and let $\mathcal {T}^*_n$ denote the set of such trees with n+1 leaves (i.e., *n* leaves not counting the leftmost child of the root). A tree $T\in \mathcal {T}^*$ is commonly called a *planted tree*. We take trees in $\mathcal {T}^*$ to be labeled so that vertices in even generations have label *P* and vertices in odd generations have label *S*. There is a useful bijection

**Fig. 4:**
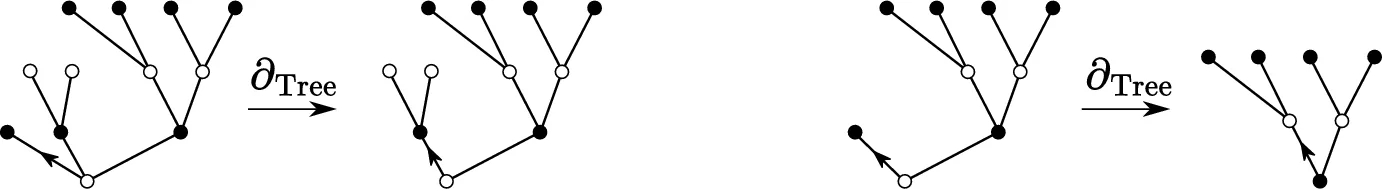
The removal of the first child of the root. Here, the vertices ∘ correspond to label *P* and the vertices ∙ to label *S*

2.4 $$\begin{aligned} \partial _{\textrm{Tree}}: \mathcal {T}^*\rightarrow \mathcal {T}{\mathcal {N}}, \end{aligned}$$

which is described as follows. Let $T\in \mathcal {T}_{n}^*$. If the outdegree of the root in *T* is strictly greater than 2, remove the leftmost child of the root (along with the edge adjacent to it). Note that the resulting tree is still labeled by *P*, so this yields an element in $\mathcal {T}\mathcal {P}_n$. If the outdegree of the root in *T* equals 2, then remove the root and its leftmost child along with their adjacent edges. The resulting tree is labeled by *S* and is thus an element in $\mathcal {T}\mathcal {S}_n$, see Fig. 4. In the case when *T* has exactly 1 leaf, let $\partial _\textrm{Tree}T = \bullet$.

### Bijection Between SP Maps and Labeled Trees

Both SP networks N (2.1) and labeled trees $\mathcal{T}\mathcal{N}$ (2.3) may be described in the language of combinatorial species [14]. This provides a natural framework for defining bijections $\varphi :\mathcal {T}\mathcal {N}\rightarrow \mathcal {N}$ and $\varphi ^*: \mathcal {T}^*\rightarrow \mathcal{S}\mathcal{P}$, as we now describe.

First, an element of N is either the single edge $e = (*_0,*_1)$, or a parallel network or a series network:

2.5 $$\begin{aligned} \mathcal {N}&= e + \mathcal {P}+ \mathcal {S}. \end{aligned}$$

The elements of $\mathcal {P}$ are called parallel networks and are constructed from a parallel composition of at least two non-parallel networks:

2.6 $$\begin{aligned} \mathcal {P}&= {\textsc {SEQ}}_{\ge 2} (\mathcal {N}- \mathcal {P}), \end{aligned}$$

where the combinatorial species ${\textsc {SEQ}}_{\ge 2}$ are the linear orders of length greater than or equal to two. The elements of $\mathcal {S}$ are called series networks and are constructed from a series composition of at least two non-series networks:

2.7 $$\begin{aligned} \mathcal {S}&= {\textsc {SEQ}}_{\ge 2} ( \mathcal {N}\,-\, \mathcal {S}). \end{aligned}$$

The single edge network is treated separately in the above definitions since it is in some sense neither series nor parallel.

Similarly, a tree from $\mathcal {T}\,{\mathcal {N}}$ is either the unlabeled single leaf ∙ or a tree labeled by *P* or a tree labeled by *S*:

2.8 $$\begin{aligned} \mathcal {T}\,{\mathcal {N}}&= \bullet + \mathcal {T}\,\mathcal {P}+ \mathcal {T}\,\mathcal {S}. \end{aligned}$$

A tree labeled by *P* is constructed by a composition of at least 2 trees from $\mathcal {T}_{\mathcal {N}}$, which are not labeled by *P*:

2.9 $$\begin{aligned} \mathcal {T}\,\mathcal {P}&= {\textsc {SEQ}}_{\ge 2} ( \mathcal {T}\,{\mathcal {N}} - \mathcal {T}\,\mathcal {P}). \end{aligned}$$

A tree labeled by *S* is constructed by a composition of at least 2 trees from $\mathcal {T}_{\mathcal {N}}$, which are not labeled by *S*:

2.10 $$\begin{aligned} \mathcal {T}\,\mathcal {S}&= {\textsc {SEQ}}_{\ge 2} (\mathcal {T}\,{\mathcal {N}} - \mathcal {T}\,\mathcal {S}). \end{aligned}$$

The combinatorial identities (2.5), (2.6), (2.7) and (2.8), (2.9), (2.10) naturally define a bijection

$$\begin{aligned} \varphi :\mathcal {T}\mathcal {N}\rightarrow \mathcal {N}. \end{aligned}$$

**Fig. 5:**
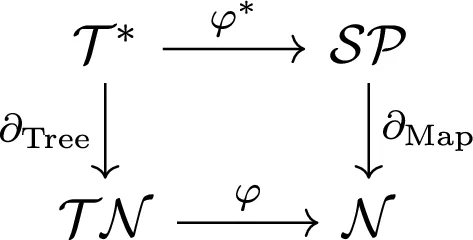
Definition of $\varphi ^*$ from φ

Moreover, networks with *n* edges are in bijection with the set of labeled trees with *n* leaves and networks in $\mathcal {P}$ and $\mathcal {S}$ are in bijection with labeled trees in $\mathcal {T}\,\mathcal {P}$ and $\mathcal {T}\,\mathcal {S}$, respectively. Combining φ with the bijections (2.2) and (2.4) allows us to define a bijection $\varphi ^*: \mathcal {T}^*\rightarrow \mathcal{S}\mathcal{P}$ as in Fig. 5.

It will be useful to have the following recursive description of $\varphi ^*$. Given a labeled tree $(T,\ell )\in \mathcal {T}\times \{S,P\}$, first assign a single edge to each of the leaves. Then, start forming networks by combining the edges corresponding to sibling leaves in series or in parallel, as determined by the label of their common parent. Proceeding recursively toward the root of the tree, each vertex corresponds to a series or a parallel network, which is constructed from the subtree of its descendants. Networks corresponding to vertices with a common parent are connected in series if that parent has label *S*, respectively, in parallel if it has label *P*. To be definite, parallel connections are made in the same order left-to-right as the order of the corresponding siblings, while series connections are made so that the left-to-right order of the siblings gives the bottom-to-top order of connections. Restricting this construction to $\mathcal {T}^*$ gives the bijection $\varphi ^*$.

### Blobs

**Fig. 6:**
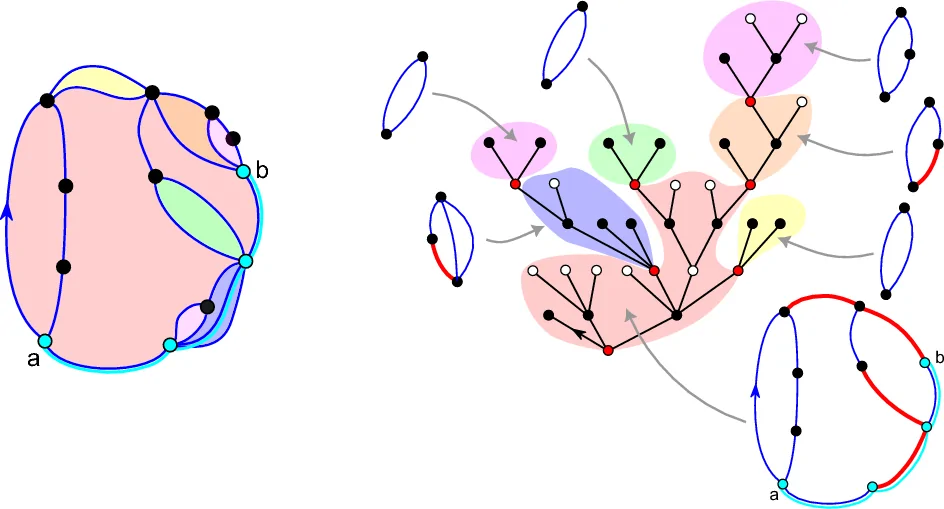
Left: An example of a rooted, two-connected SP map divided into blobs. A geodesic is traced from *a* to *b* (in cyan). Right: Its corresponding planted plane tree, labeled by *P*, divided into segments. Here, ∘ and  denote the label *P* and ∙ denotes the label *S*. Each segment corresponds to a network, and the red edges depicted in these networks are the places where networks corresponding to fringe subtrees are inserted

We now define what we call *blobs*. Informally, blobs are parts of *M* that are separated by bottlenecks, in the sense that a geodesic between two vertices within the same blob has all its vertices inside that blob (see Fig. 6 for an illustration of the description that follows).

Suppose that we are given a rooted two-connected SP map $M\in \mathcal {S}\mathcal {P}$ and the corresponding planted tree $T^*=(\varphi ^*)^{-1}(M)\in \mathcal {T}^*$ (which we recall is labeled by *P*). We color a vertex of $T^*$ red if it is marked *P* and has at least one child that is a leaf. In particular, the root of $T^*$ is colored red. This way, the tree $T^*$ is decomposed into (maximal) subtrees whose roots are red, and where any red non-root vertex is a leaf. We call these subtrees *segments*.

We define the *blobs* of *M* to be the networks obtained by applying the recursive construction defining $\varphi ^*$ (described at the end of Sect. 2.3) to the segments. We moreover declare the blobs to have an oriented red placeholder edge for each red non-root vertex in the segment, which marks the location where we would insert the network constructed from the corresponding fringe subtree in $T^*$.

The orientation of each red edge is naturally inherited from the orientation of the edge in the tree from the root to the red vertex, together with the orientation of the root edge of the SP map, where the tree is explored in its usual lexicographic order. We assign placeholder south and north poles to each of the blobs as the start- and endpoints of the oriented edge that corresponds to the red edge assigned to the red leaf of the corresponding parent segment. This way, the map *M* gets decomposed into blobs, where the placeholder poles of each blob are connected by an edge in *M*.

We perform a similar decomposition of networks *N* into blobs, where all definitions are analogous except that we demand the root vertex of the corresponding tree *T* to be colored red.

### Bienaymé–Galton–Watson Trees and Simply Generated Trees

The main focus of this work is uniformly distributed two-connected SP maps. However, the bijective correspondence with trees that is explained in the previous section allows us easily to consider a more general measure on the maps by assigning weights to the trees in a natural way and pushing these forward to the maps using the bijection.

Let $\mu = (\mu (k))_{k\ge 0}$ be a probability measure with μ(1)=0. Let $\operatorname {BGW}^\mu$ denote the distribution of a Bienaymé–Galton–Watson process with offspring distribution μ. If μ has mean less than or equal to 1, then the tree τ is almost surely finite and, for any finite tree *T*,

2.11 $$\begin{aligned} \operatorname {BGW}^{\mu }(\tau =T) = \prod _{u \in T}\mu (\operatorname {out}_\tau (u)). \end{aligned}$$

Let $\operatorname {BGW}^{\mu }_n$ be the corresponding measure conditioned on the number of leaves being *n*. The random trees distributed by $\operatorname {BGW}^\mu$ and $\operatorname {BGW}^\mu _n$ will be denoted by $\textsf{T}^\mu$ and $\textsf{T}^\mu _n$, respectively.

In order to relate the BGW trees to the random maps, it will be useful to consider another (almost) equivalent model of random trees called *simply generated trees with leaves as atoms*. Let $\eta = (\eta (i))_{i\ge 0}$ be a sequence of nonnegative numbers called *weights* and assume that η(1)=0. Let $T\in \mathcal {T}_n$ and define the weight of *T* by

$$\begin{aligned} \eta (T) = \prod _{u\in T} \eta (\operatorname {out}_T(u)). \end{aligned}$$

A *simply generated tree* on *n* leaves is a random plane tree τ with values in $\mathcal {T}_n$ with a probability distribution

2.12 $$\begin{aligned} \mathbb {P}(\tau = T) = \frac{\eta (T)}{\sum _{T'\in \mathcal {T}_n} \eta (T')}, \qquad T \in \mathcal {T}_n. \end{aligned}$$

The literature contains many results on simply generated trees with a fixed number of vertices, and for these we refer to [13] for details. The current case, where instead the number of leaves is fixed, is considered in, e.g., [21]. Below, we outline the main results which we require.

For a,b>0, let $\eta ^{a,b}$ be another weight sequence defined by

2.13 $$\begin{aligned} \eta ^{a,b}(k)= {\left\{ \begin{array}{ll} a \eta (0) &  k = 0 \\ b^{k-1} \eta (k) &  k\ge 2. \end{array}\right. } \end{aligned}$$

These weights are equivalent to η in the sense that they define the same measure as in (2.12). Define the generating series

2.14 $$\begin{aligned} \qquad g^\eta (z) = \sum _{n=0}^\infty \eta (n) z^n \end{aligned}$$

and denote its radius of convergence by $\rho _\eta$. We will assume that the weights η are chosen such that $\rho _\eta > 0$. Then, for any b>0 such that $\frac{g^\eta (b)-\eta (0)}{\eta (0) b} < 1$, one may choose

$$\begin{aligned} a = \frac{1}{\eta (0)}-\frac{g^\eta (b)-\eta (0)}{\eta (0) b} \end{aligned}$$

in which case $\eta ^{a,b}$ is a probability measure which we will denote by $\eta ^{(b)}$. Denote its mean by

$$\begin{aligned} \textbf{m}_b = \sum _{k=2}^\infty k \eta ^{(b)}(k) = \sum _{k=2}^\infty k\eta (k) b^{k-1} = (g^\eta )'(b). \end{aligned}$$

The mean is a strictly increasing function of *b* and $\textbf{m}_0=0$. In the current work, we will assume that there is a $b<\rho _\eta$ such that $\textbf{m}_b = 1$, which is then necessarily unique. The corresponding probability measure will be denoted by μ, and the condition $\textbf{m}_b = 1$ is referred to as *criticality*. The condition $b<\rho _\eta$ furthermore guarantees that μ has finite exponential moments. It is straightforward to check that the simply generated tree with weights η has the same distribution as $\textsf{T}^\mu _n$.

### Weighted Trees and Maps

Let η be a weight sequence such that there exists a corresponding critical offspring distribution μ. Generate a random planted tree, denoted by $\textsf{T}_n^{\mu ,*}$, as follows:

1. Let $\textsf{T}_n^\mu$ have distribution $\operatorname {BGW}^\mu _n$.
2. Flip a fair coin and label $\textsf{T}_n^\mu$ by *P* or *S* according to the outcome of the coin flip.
3. Plant the resulting labeled tree, i.e., set $\textsf{T}_n^{\mu ,*}:= \partial _\textrm{Tree}^{-1}\textsf{T}_n^\mu$.

#### Definition 2.2

The random, weighted, rooted, two-connected SP map with *n* non-root edges is defined as:

$$\begin{aligned} \textsf{SP}_n^\mu := \varphi ^*(\textsf{T}^{\mu ,*}_n). \end{aligned}$$

The corresponding random network is denoted by:

$$ \textsf{N}_{n}^\mu = \partial _\textrm{Map}\textsf{SP}_n^\mu = \varphi (\textsf{T}_{n}^\mu ). $$

The networks obtained by conditioning $\textsf{N}_n^\mu$ on being a series or a parallel network (i.e., on the outcome of the coin flip above) are denoted by $\textsf{S}_n^\mu$ and $\textsf{P}_n^\mu$, respectively.

In order to lighten the notation, we will often suppress the μ and write, e.g., $\textsf{SP}_n = \textsf{SP}^\mu _n$, $\textsf{N}_n = \textsf{N}^\mu _n$, $\textsf{T}= \textsf{T}^\mu$, $\textsf{T}^*_n = \textsf{T}^{\mu ,*}_n$, and $\textsf{T}_n = \textsf{T}^\mu _n$.

#### Example 2.3

The uniformly distributed map $\textsf{SP}_n$ is obtained by choosing η(k)=1 for all k≥0, k≠1. In this case, one finds that

2.15 $$\begin{aligned} \mu (0)&= 2-\sqrt{2}, \quad \mu (1) = 0, \quad \mu (k) = \left( 1-\frac{1}{\sqrt{2}}\right) ^{k-1}, \qquad k\ge 2. \end{aligned}$$

#### Example 2.4

Let N≥2 be an integer. The map $\textsf{SP}_n$, which is distributed uniformly among those where each series and each parallel composition in the network $\textsf{N}_{n}$ consists of exactly *N* components, is obtained by choosing the weights $\eta (0) = \eta (N)$ and all other weights equal to 0. In the corresponding labeled tree $\textsf{T}_{n}$, every vertex has outdegree 0 or *N* and the root has outdegree 1 or *N*. In this case, one finds that $\mu (0) = 1-\frac{1}{N}$, $\mu (N) = \frac{1}{N}$.

## Proofs of the Main Results

We now turn to the proofs of Theorems 1.1 and 1.2. The main method is to compare lengths of geodesics between vertices in a series–parallel map and between corresponding vertices in its associated tree. For concreteness, we will work with the random parallel networks $\textsf{P}_n$. The associated tree is then a BGW tree $\textsf{T}_n=\textsf{T}^\mu _n$ labeled by *P*. The proof can easily be adapted to the maps $\textsf{S}_n$, $\textsf{N}_n$, and $\textsf{SP}_n$ with exactly the same outcome.

Let $\textsf{T}= \textsf{T}^\mu$ be an unconditioned BGW tree and let ξ be a random variable with distribution μ. We will use the *local limit* $\hat{\textsf{T}}$ of $\textsf{T}_n$ (as $n\rightarrow \infty$), which is an infinite random tree with a unique half-infinite path starting from the root, called its *spine*. In $\hat{\textsf{T}}$ non-spine vertices have offspring according to independent copies of ξ, whereas spine vertices have offspring according to independent copies of the size-biased random variable $\hat{\xi }$ with distribution $\mathbb {P}(\hat{\xi }=k) = k \mathbb {P}(\xi = k)$, and the successor spine vertex is chosen uniformly at random among these children. For further details, we refer to Janson’s extensive review [13].

For each $\ell \ge 1$, let $\textsf{T}^{(\ell )}$ denote the tree obtained by deleting all descendants of the (ℓ+1)st spine vertex of $\hat{\textsf{T}}$ and identifying the (ℓ+1)st spine vertex with the root of an independent copy of $\textsf{T}$. We view all the random trees $\textsf{T}_n$, $\textsf{T}$, $\hat{\textsf{T}}$, and $\hat{\textsf{T}}^{(\ell )}$ for $\ell \ge 1$ as being colored (i.e., some vertices are red) and labeled by *P* or *S* according to the fair coin flip as defined in Sect. 2.

We may define infinite random series–parallel maps/networks $\textsf{SP}$, $\textsf{N}$, $\textsf{P}$ and $\textsf{S}$ in a natural way by extending the functions φ and $\varphi ^*$ to infinite trees. The maps/networks may then be viewed as the weak local limits of the corresponding sequences of finite maps/networks. These infinite objects are not strictly needed in the following arguments. However, it is convenient to refer to them occasionally, so for completeness, we provide some details of their construction in the appendix.

We may generate $\textsf{T}$ by starting with a root segment and a list of i.i.d. non-root segments. Likewise, $\hat{\textsf{T}}$ can be generated from a root segment, a list of i.i.d. normal non-root segments, and a list of i.i.d. spine non-root segments. The tree $\hat{\textsf{T}}^{(\ell )}$ additionally has a mixed segment containing the tip of the spine.

### Lemma 3.1

The number of vertices in each type of segment (root, spine, and normal) has finite exponential moments.

### Proof

The number of vertices in each random segment may be stochastically bounded by two times the total population of a subcritical branching process (with possibly a modified root degree) whose offspring distribution is light-tailed. The total population of such a process is light-tailed itself by [13, Thm. 18.1].

We detail this argument for the root segment of $\textsf{T}^\mu$. The proofs for all other types of segments are analogous. We construct a BGW tree $\textsf{T}^\nu$ with offspring probabilities $(\nu _n)_n$ from $\textsf{T}^\mu$ as follows. Let $\textsf{T}'$ be the root segment of $\textsf{T}^\mu$. The tree $\textsf{T}^\nu$ is defined as the tree with vertex set consisting of the vertices in even generations in $\textsf{T}'$ and vertices in $\textsf{T}^\nu$ are connected by an edge if and only if one is the grandchild of the other in $\textsf{T}'$. Note that since $\textsf{T}'$ has no vertices of outdegree 1, the number of vertices in $\textsf{T}'$ is less than two times the number of its leaves and $\textsf{T}^\nu$ has the same number of leaves as $\textsf{T}'$. Therefore, the number of vertices in $\textsf{T}'$ is bounded by two times the number of leaves in $\textsf{T}^\nu$.

Another way to describe $\textsf{T}^\nu$ is the following. Let *v* be a vertex in $\textsf{T}^\mu$ at an even generation whose children we have not yet generated and assume that *v* is also present in $\textsf{T}^\nu$. If *v* either has no children in $\textsf{T}^\mu$ or it has more than one child and some of its children have no children, then we say that *v* has no children in $\textsf{T}^\nu$. On the other hand, if *v* has children in $\textsf{T}^\mu$ and all of its children also have children, then we add these grandchildren as the children of *v* in $\textsf{T}^\nu$. From this description, we find that

$$\begin{aligned} \nu (0)&= 1-\sum _{n=2}^\infty \mu (n) (1-\mu (0))^n, \\ \nu (1)&= 0, \\ \nu (k)&= \sum _{n=2}^\infty \mu (n) \sum _{i_1+\cdots + i_n = k}\prod _{j=1}^n \mu (i_j)1\!\!1\{i_j > 0\}, \quad k\ge 2. \end{aligned}$$

The generating function *g* of the probabilities $(\nu _n)_n$ satisfies

3.1 $$\begin{aligned} g(z) = \nu (0) + h(h(z)) \end{aligned}$$

where $h(z) = f(z) - \mu _0$ and *f* is the generating function of the sequence $(\mu _n)_n$. We then have

$$\begin{aligned} g'(1) = h'(h(1))h'(1) = h'(1-\mu (0)) = f'(1-\mu (0)) < 1 \end{aligned}$$

since $f^{\prime }\left(1\right)=1$. Thus, the ν-process is subcritical.

Since μ has finite exponential moments, we find that ν also has finite exponential moments. Thus, the total population of $\textsf{T}^\nu$ (and hence also the size of the root segment of $\textsf{T}$) has finite exponential moments by [13, Thm. 18.1]. □

### Corollary 3.2

There are constants C,c>0 such that the probability for the maximal size of a segment in $\textsf{T}_n$ to be larger than *x* is bounded by $C n^{5/2} \exp (-cx)$ uniformly for all n≥2 and x>0.

### Proof

The probability for $\textsf{T}$ to have *n* leaves satisfies

3.2 $$\begin{aligned} \mathbb {P}\left( |\textsf{T}|=n\right) \sim c_{\textrm{cond}} n^{-3/2} \end{aligned}$$

for some constant $c_{\textrm{cond}}>0$, see, for example,  [15]. Let $(X_i)_i$ denote the sizes of the segments in *T*. There are at most *n* segments in $\textsf{T}_n$; hence, the probability for the maximal size of a segment in $\textsf{T}_n$ to be larger than x>0 satisfies

$$\begin{aligned} \mathbb {P}\left( \max _i X_i> x~\Big |~ |\textsf{T}|=n\right)&= \frac{\mathbb {P}\left( \bigcup _i\{X_i> x\}, |\textsf{T}|=n\right) }{\mathbb {P}\left( |\textsf{T}|=n\right) } \\&\le c_{\textrm{cond}}^{-1} n^{3/2} \sum _{i=1}^n \mathbb {P}(X_i > x), \end{aligned}$$

where |*T*| denotes the number of leaves in *T*. By Lemma 3.1, each segment of the unconditioned tree has a finite exponential moment and the segments are identically distributed, except possibly the root segment. Thus, each term in the sum is bounded by a constant times $e^{-cx}$, for some c>0, and the result follows. □

In the infinite map $\textsf{P}$ corresponding to the tree $\hat{\textsf{T}}$, the blobs $B_0, B_1, \ldots$ corresponding to the spine segments are called *spine blobs*. The spine blobs are independent, and the non-root spine blobs $(B_i)_{i \ge 1}$ are identically distributed. For each $\ell \ge 1$, consider the graph distance $X_\ell$ from the south pole of $B_0$ to the south pole of $B_\ell$.

### Lemma 3.3

There exists a constant η>0 such that for each ϵ>0, there are constants C,c>0 with

$$ \mathbb {P}(|X_\ell - \eta \ell | > \epsilon \ell ) \le C \exp (-c\ell ) $$

uniformly for all $\ell \ge 1$.

### Proof

Consider the graph distance $Y_\ell$ from the south pole *v* of $B_1$ to the south pole of $B_\ell$. This way, $|X_\ell - Y_\ell |$ is bounded by the size of $B_0$, which has finite exponential moments by Lemma 3.1. It hence suffices to show that there exists a constant η>0 such that for each ϵ>0, there are constants C,c>0 such that for all $\ell \ge 1$,

3.3 $$\begin{aligned} \mathbb {P}(|Y_\ell - \eta \ell | > \epsilon \ell ) \le C \exp (-c\ell ). \end{aligned}$$

Since the poles of $B_{\ell }$ are joined by an edge, the graph distances from *v* to either of them (north or south) differ by at most 1. Hence, the increment $Y_{\ell +1} - Y_{\ell }$ only depends on this difference $S_\ell \in \{-1,0,1\}$ and $B_{\ell }$. Thus, $(Y_{\ell }, S_\ell )_{\ell \ge 1}$ is a Markov-additive process with a driving chain $(S_\ell )_{\ell \ge 1}$ whose state space has three elements. Inequality (3.3) now immediately follows from a general large deviation result for Markov-additive processes [12, Thm. 5.1] whose conditions are satisfied since the state space of the driving chain under consideration is finite [12, Rem. 3.5, Sec 7 (ii)]. □

### Remark 3.4

We leave out details concerning the exact transition probabilities of the Markov-additive process in the above proof but refer to the paper by Curien, Haas, and Kortchemski [9] for a similar situation. In our case, the increments of the additive component $(Y_\ell )_{\ell \ge 1}$ of the chain are not as explicit as in [9] (they are given in terms of distances between certain vertices in a spine blob), and therefore, it is less useful to write them down.

### Lemma 3.5

Let $Z_\ell$ be the number of segments encountered on the spine from the root of $\hat{\textsf{T}}$ to the (ℓ+1)st spine vertex. There exists a constant κ>0 such that for each ϵ>0, there are constants C,c>0 such that for all $\ell \ge 1$,

$$ \mathbb {P}(|Z_\ell - \kappa \ell | > \epsilon \ell ) \le C \exp (-c\ell ). $$

### Proof

Let $E_m$ denotes the number of edges on the spine we need to cross in order to get from the root vertex to the start of the (m+1)st segment. Set $E_0=0$. Hence, the differences $E_m - E_{m-1}$ are independent for $\ell \ge 1$ and identically distributed for all m≥2. These increments have a geometric distribution, since they may be identified by a sequence of independent Bernoulli coin flips.

We make use of the following inequality given, for example, in [18, Example 1.4]: Let $S_n$ denotes the sum of *n* i.i.d. real-valued centered random variables with finite exponential moments. Then, there are constants $\lambda _0, c>0$ such that for all $n\in \mathbb {N}$, x>0 and $0\le \lambda \le \lambda _0$ it holds that

3.4 $$\begin{aligned} \mathbb {P}(|S_n| \ge x) \le 2 \exp (c n \lambda ^2 - \lambda x). \end{aligned}$$

It follows that there exists $\kappa ^{-1}>0$ such that for each δ>0, there exist constants C,c>0 with

$$ \mathbb {P}(|E_m - \kappa ^{-1} m| > \delta m) \le C \exp (-c m), \qquad \text {for all } m\ge 1. $$

As δ>0 is arbitrary, it follows by choosing $$\delta = \pm \left( 1-\frac{1}{1\pm \epsilon }\right) \kappa ^{-1}$$ that for any ϵ>0, there exist constants $C^{\prime },c^{\prime }>0$ with

$$ \mathbb {P}(\ell \notin [ E_{\lfloor \kappa \ell (1-\epsilon ) \rfloor }, E_{\lfloor \kappa \ell (1+\epsilon ) \rfloor }] )\le C' \exp (-c'\ell ). $$

Since $\ell \notin [ E_{\lfloor \kappa \ell (1-\epsilon ) \rfloor }, E_{\lfloor \kappa \ell (1+\epsilon ) \rfloor }$ is equivalent to $|Z_\ell - \kappa \ell | > \kappa \epsilon \ell$, the proof is complete. □

Before proceeding with the proof, we recall some facts about the Gromov–Hausdorff metric $d_{\textrm{GH}}$ on the space of isometry classes of compact metric spaces. Informally, two compact metric spaces are close in the Gromov–Hausdorff metric $d_{\textrm{GH}}$ if they may be isometrically embedded in the third space *Z* so that their Hausdorff distance in *Z* is small. Here, we will use a representation of $d_{\textrm{GH}}$ in terms of distortions of correspondences, see, e.g.,  [7]. A correspondence $\mathcal {R}$ between two metric spaces $(A,d_A)$ and $(B,d_B)$ is a subset $\mathcal {R}\subseteq A \times B$ such that for any a∈A, there is a b∈B such that $(a,b)\in \mathcal {R}$, and for any b∈B, there is an a∈A such that $(a,b)\in \mathcal {R}$. A distortion of a correspondence $\mathcal {R}$ is defined by:

$$\begin{aligned} \operatorname {dis}(\mathcal {R}) = \sup \{|d_A(a_1,a_2)-d_B(b_1,b_2)|~:~(a_1,b_1),(a_2,b_2)\in \mathcal {R}\}. \end{aligned}$$

The Gromov–Hausdorff metric may then be written as:

$$\begin{aligned} d_{\textrm{GH}}(A,B) = \frac{1}{2} \inf _\mathcal {R}\operatorname {dis}(\mathcal {R}). \end{aligned}$$

By the main theorem of [15, 19], there exists a constant $c_{\textrm{tree}}>0$ such that

3.5 $$\begin{aligned} (\textsf{T}_n, c_{\textrm{tree}} n^{-1/2} d_{\textsf{T}_n}) \,{\buildrel d \over \longrightarrow }\,(\mathcal {T}_{\textrm{e}}, d_{\mathcal {T}_{\textrm{e}}}) \end{aligned}$$

in the Gromov–Hausdorff topology, with $(\mathcal {T}_{\textrm{e}}, d_{\mathcal {T}_{\textrm{e}}})$ denoting Aldous’ Brownian tree. The rest of the proof involves defining a suitable correspondence between the spaces $\textsf{P}_n$ and $\textsf{T}_n$ and showing that the distortion of that correspondence, with properly rescaled metrics, converges to 0 in probability.

We define a correspondence $\textsf{R}_n$ between $\textsf{P}_n$ and $\textsf{T}_n$ as follows. Note that there is a bijective relationship between blobs in $\textsf{P}_n$ and segments in $\textsf{T}_n$. We let $(u,v) \in \textsf{R}_n$ if and only if there is a blob in $\textsf{P}_n$, which contains *u* and a corresponding segment in $\textsf{T}_n$ which contains *v*. In that case, we write u∼v.

Let $\textrm{h}_{\textsf{P}_n}(u)$ denotes the graph distance in the map $\textsf{P}_n$ from the south pole $*_0$ to *u*, and $\textrm{h}_{\textsf{T}_n}(v)$ the height of the vertex *v* in $\textsf{T}_n$. The following lemma is the first step in relating distances in $\textsf{P}_n$ to distances in $\textsf{T}_n$.

### Lemma 3.6

Let η be the constant in Lemma 3.3, κ the constant in Lemma 3.5, and let δ>0 be arbitrary. With probability tending to one as $n \rightarrow \infty$, there exist no vertices $u \in \textsf{P}_n$ and $v \in \textsf{T}_n$ such that u∼v and

3.6 $$\begin{aligned} |\textrm{h}_{\textsf{P}_n}(u) - \eta \kappa \textrm{h}_{\textsf{T}_n}(v)| \ge \delta \sqrt{n}. \end{aligned}$$

### Proof

Let ϵ>0 be given. Let $H_n = H\sqrt{n}$ for some H>0. By (3.5), we may choose *H* sufficiently large such that the height $\textrm{H}(\textsf{T}_n)$ satisfies

$$ \mathbb {P}(\textrm{H}(\textsf{T}_n) > H_n) \le \epsilon $$

for all *n*. Let us call a vertex *v* in a tree τ *bad* if it corresponds to some vertex *u* in the associated map *P* such that Inequality (3.6) holds (with obvious replacements of $\textsf{P}_n$ and $\textsf{T}_n$ by *P* and τ). Let $\mathcal {E}(\tau )$ denote the property that a tree *T* contains a bad vertex. Let us denote the tip of the spine in $\hat{\textsf{T}}^{(\ell )}$ by $U_\ell$. Given any finite plane tree *T* with a vertex *u* having height ℓ, it holds that

$$\begin{aligned} \mathbb {P}\left( (\hat{\textsf{T}}^{(\ell )},U_\ell )=(T,u)\right) = \mathbb {P}\left( \textsf{T}= T\right) \end{aligned}$$

(see, e.g.,  the short derivation in [20, Appendix A3]). Hence, using (3.2), we obtain

$$\begin{aligned} \mathbb {P}(\mathcal {E})&\le \epsilon + \mathbb {P}(\mathcal {E}(T_n), \textrm{H}(\textsf{T}_n) \le H_n) \\&\le \epsilon + \frac{2n }{\mathbb {P}(|\textsf{T}|=n)} \sum _{\ell =0}^{\lfloor H_n \rfloor } \mathbb {P}(U_\ell \text { is bad}) \\&= \epsilon + O(n^{5/2}) \sum _{\ell =0}^{\lfloor H_n \rfloor } \mathbb {P}(U_\ell \text { is bad}). \end{aligned}$$

The second inequality is explained as follows. We may write:

$$\begin{aligned}&\mathbb {P}(\mathcal {E}(T_n), \textrm{H}(\textsf{T}_n) \le H \sqrt{n})\mathbb {P}(|\textsf{T}|=n) = \sum _{\tau \in \mathcal {T}} {1\!\!1}\{\mathcal {E}(\tau ),\textrm{H}(\tau )\le H_n,|\tau |=n\} \mathbb {P}(\textsf{T}= \tau ) \\&\le \sum _{\tau \in \mathcal {T}, u\in \tau } {1\!\!1}\{u \text { is bad},\textrm{H}(\tau )\le H_n,|\tau |=n\} \mathbb {P}(\textsf{T}= \tau ) \\&= \sum _{\tau \in \mathcal {T}, u\in \tau }\sum _{\ell =0}^{\textrm{H}(\tau )} {1\!\!1}\{u \text { is bad},\textrm{H}(\tau )\le H_n,|\tau |=n,\textrm{h}_{\tau }(u)=\ell \} \mathbb {P}\left( (\hat{\textsf{T}}^{(\ell )},U_\ell )=(\tau ,u)\right) \\&\le 2n \sum _{\ell = 0}^{\lfloor H_n \rfloor }\sum _{\tau \in \mathcal {T}} \mathbb {P}\left( \hat{\textsf{T}}^{(\ell )} = \tau ,U_\ell \text { is bad}\right) = 2n \sum _{\ell = 0}^{\lfloor H_n \rfloor } \mathbb {P}\left( U_\ell \text { is bad}\right) . \end{aligned}$$

For the last inequality, we used the fact that if a plane tree has *n* leaves and no inner vertex with exactly one child, then it has at most 2*n* vertices in total.

If *u* is a vertex in the map corresponding to $\hat{\textsf{T}}^{(\ell )}$ such that *u* corresponds to the tip of the spine of $\hat{\textsf{T}}^{(\ell )}$, then the height of *u* may be bounded by the total number of leaves in the root segments of the subtrees attached to the spine vertices of $\hat{\textsf{T}}^{(\ell )}$. Denote by $W_i$ the total size of all the root segments in the subtrees attached to the spine vertex at distance *i* from the root, where $0 \le i \le \ell$. Note that they are independent and i.i.d for i<ℓ. At each of these spine vertices, the total number of subtrees is distributed as $\hat{\xi }-1$ or $\hat{\xi }$ (at the tip of the spine) and is thus light-tailed. Furthermore, the total size of the root segment of each of the subtrees is light-tailed by Lemma 3.1. We conclude that $W_i$ is light-tailed for all $0 \le i \le \ell$.

If $\ell <\delta / (2 \eta \kappa ) \sqrt{n}$, then the event that $U_\ell$ is bad implies that the height of *u* is larger than $\delta \sqrt{n}$ and, therefore, that

$$\begin{aligned} \sum _{i=0}^\ell W_i \ge \delta \sqrt{n}. \end{aligned}$$

Since the $W_i$ is i.i.d and light-tailed, it follows by the medium deviation Inequality (3.4) that if we take h>0 small enough, then there exist constants $C_1, c_1>0$ such that uniformly for all *n* and $1 \le \ell \le h\sqrt{n}$,

3.7 $$\begin{aligned} \mathbb {P}(U_\ell \text { is bad}) \le \mathbb {P}\left( \sum _{i=0}^{h\sqrt{n}} W_i \ge \delta \sqrt{n}\right) \le C_1 \exp (-c_1 \sqrt{n}). \end{aligned}$$

This ensures

$$ O(n^{5/2}) \sum _{\ell =0}^{\lfloor h \sqrt{n} \rfloor } \mathbb {P}(U_\ell \text { is bad}) = O(n^3) \exp (-c_1 \sqrt{n}) = \exp (-\Theta (\sqrt{n})). $$

Furthermore, uniformly for $h \sqrt{n} \le \ell \le H \sqrt{n}$, we know by Lemma 3.5 that for any c>0, we have $|Z_\ell - \kappa \ell | \le c \ell$ with probability at least $1 - \exp (\Theta (\sqrt{n}))$, which allows us to apply Lemma 3.3 to obtain for any c>0 that $|X_{Z_\ell } - \eta \kappa \ell | \le c \ell$ again with probability at least $1 - \exp (\Theta (\sqrt{n}))$. Recall that we constructed $\hat{\textsf{T}}^{(\ell )}$ from $\hat{\textsf{T}}$ by replacing the descendants of the tip of the spine, therefore creating a “mixed” segment that contains the tip of the spine. Hence, in the map corresponding to $\hat{\textsf{T}}^{(\ell )}$, any vertex corresponding to the tip of the spine has a height that differs from $X_{Z_\ell }$ at most by a summand that by Lemma 3.1 has finite exponential moments. Thus, we obtain:

$$ O(n^{5/2}) \sum _{h \sqrt{n} \le \ell \le H \sqrt{n}} \mathbb {P}(U_\ell \text { is bad}) = O(n^3) \exp (-\Theta (\sqrt{n})) = \exp (-\Theta (\sqrt{n})). $$

In summary,

$$ \mathbb {P}(\mathcal {E}) \le \epsilon + \exp (-\Theta (\sqrt{n})). $$

Since ϵ>0 was arbitrary, this completes the proof. □

We are now ready to prove our main theorems.

### Proof of Theorem 1.1

By Corollary 3.2 and Lemma 3.6, we know that, for some constant C>0 and some deterministic sequence $t_n =o(\sqrt{n})$, we have with high probability that the largest blob in $\textsf{P}_n$ has diameter at most Clogn, and whenever a vertex *u* in $\textsf{P}_n$ corresponds to a vertex *v* in $\textsf{T}_n$, then

3.8 $$\begin{aligned} |\textrm{h}_{\textsf{P}_n}(u) - \eta \kappa \textrm{h}_{\textsf{T}_n}(v)| \le t_n. \end{aligned}$$

Now, let $u_1, u_2$ be vertices in $\textsf{P}_n$ and let $v_1$ and $v_2$ be any vertices in $\textsf{T}_n$ such that $v_i$ corresponds to $u_i$ for i=1,2. If $v_3$ denotes the lowest common ancestor of $v_1$ and $v_2$ in $\textsf{T}_n$, then

$$ d_{\textsf{T}_n}(v_1, v_2) = \textrm{h}_{\textsf{T}_n}(v_1) + \textrm{h}_{\textsf{T}_n}(v_2) - 2\textrm{h}_{\textsf{T}_n}(v_3). $$

Any geodesic in $\textsf{P}_n$ between $u_1$ and $u_2$ must pass through a blob corresponding to $v_3$. Letting $u_3$ denotes some vertex in this blob, it follows that:

$$ d_{\textsf{P}_n}(u_1, u_2) = \textrm{h}_{\textsf{P}_n}(u_1) + \textrm{h}_{\textsf{P}_n}(u_2) - 2\textrm{h}_{\textsf{P}_n}(u_3) + O(\log n). $$

By Inequality (3.8), it follows that:

$$ d_{\textsf{P}_n}(u_1, u_2) = \eta \kappa d_{\textsf{T}_n}(v_1, v_2) + o(\sqrt{n}). $$

Thus, the correspondence between $\frac{1}{\eta \kappa }d_{\textsf{P}_n}$ and $d_{\textsf{T}_n}$ has distortion $o(\sqrt{n})$. By Equation (3.5), it follows that"

3.9 $$\begin{aligned} \left( \textsf{P}_n, \frac{c_{\textrm{tree}}}{\eta \kappa } n^{-1/2} d_{\textsf{P}_n}\right) \,{\buildrel d \over \longrightarrow }\,(\mathcal {T}_{\textrm{e}}, d_{\mathcal {T}_{\textrm{e}}}) \end{aligned}$$

in the Gromov–Hausdorff topology. □

### Proof of Theorem 1.2

Since the diameter is bounded by twice the height with respect to the origin of the root edge, it suffices to show that there exists C,c>0 with

$$ \mathbb {P}(\textrm{H}(\textsf{P}_n) > x\sqrt{n}) \le C \exp (-c x^2 ). $$

By possibly adjusting the constants *C*, *c*, it suffices to show this inequality for all x≥1. Furthermore, the left-hand side equals to zero if $x \ge \sqrt{n}$. Hence, it suffices to treat the case $1 \le x \le \sqrt{n}$.

We now use the same notation and argue analogously as in the proof of Lemma 3.6, with the exception that we call a vertex *v* in $\textsf{T}_n$ *bad* if it corresponds to some vertex *u* in $\textsf{P}_n$ such that its height in $\textsf{P}_n$ is larger than $x \sqrt{n}$. Analogously, we define for any $\ell \ge 1$ whether the tip of the spine of $\hat{\textsf{T}}^{(\ell )}$ is *bad*, and we let $\mathcal {E}$ denotes the event that $\textsf{T}_n$ contains a bad vertex.

By [21, Lem. 6.61], there exists $C_1, c_1>0$ with

$$ \mathbb {P}(\textrm{H}(\textsf{T}_n) > y \sqrt{n}) \le C_1 \exp (-c_1 y^2 ) $$

for any y>0. Hence, it follows as in the proof of Lemma 3.6 that for any fixed h>0

$$\begin{aligned} \mathbb {P}(\mathcal {E})&\le \mathbb {P}(\textrm{H}(\textsf{T}_n) > (x/h) \sqrt{n})+ \mathbb {P}(\mathcal {E}, \textrm{H}(\textsf{T}_n) \le (x/h) \sqrt{n}) \\&\le C_1 \exp (-c_1 x^2/h^2) + O(n^{5/2}) \sum _{\ell =0}^{\lfloor (x/h) \sqrt{n} \rfloor } \mathbb {P}(U_\ell \text { is bad}). \end{aligned}$$

Furthermore, applying Inequality (3.4) as before yields that there exist constants $\lambda _0, \mu _{\textrm{blob}}, c_2>0$ such that for all $n\in \mathbb {N}$ and $0 \le \lambda \le \lambda _0$ it holds that

$$\begin{aligned} \mathbb {P}(U_\ell \text { is bad}) \le 2 \exp (c \ell \lambda ^2 - \lambda (x \sqrt{n} - \ell \mu _{\textrm{blob}})). \end{aligned}$$

As h>0 was fixed but arbitrary, we may take *h* large enough such that $\mu _{\textrm{blob}} / h < 1/2$. As x≥1, it follows that uniformly for $\ell \le (x/h)\sqrt{n}$:

$$ c \ell \lambda ^2 - \lambda (x \sqrt{n} - \ell \mu _{\textrm{blob}}) \le c \ell \lambda ^2 - \lambda x \sqrt{n} /2 = -x \Theta (\sqrt{n}). $$

Hence,

$$ \mathbb {P}(\textrm{H}(\textsf{P}_n) > x\sqrt{n}) = \mathbb {P}(\mathcal {E}) \le C_1 \exp (-c_1 x^2/h^2) + \exp (-x \Theta (\sqrt{n})). $$

Since we assumed $1\le x\le \sqrt{n}$, it follows that:

$$ \mathbb {P}(\textrm{H}(\textsf{P}_n) > x\sqrt{n}) \le C_3 \exp (-c_3 x^2) $$

for some constants $C_3, c_3 >0$ that do not depend on *n*. □

## Conclusions and Related Classes

Our results imply, in particular, a scaling limit of uniformly sampled two-connected rooted series–parallel maps. From a different viewpoint, it has been shown in [5] that rooted series–parallel maps are in bijection with permutations avoiding the patterns 2413 and 3142. As a side result, it was also shown that a bipolar planar map admits a unique bipolar orientation precisely when it is series–parallel. These observations link series–parallel maps to the large framework of separable permutations and their connections to the Liouville quantum gravity, which has enjoyed remarkable developments during the last few years. For example, it is conjectured in [6] that conformally embedded rooted series–parallel maps converge toward the 2-LQG quantum sphere. Therefore, we expect interesting research directions following from our work.

Apart from planar maps, we may also consider planar graphs subject to constraints. The terminology for graph classes is a bit at odds with the one used here for planar maps. The class of series–parallel graphs refers to (simple) graphs that do not admit the $K_4$ as a minor, which may very well be separable (that is, not two-connected). This class is an important example of a subcritical graph class and hence admits the Brownian tree as scaling limit by [17].

## Figures and Tables

**Fig. 7:**
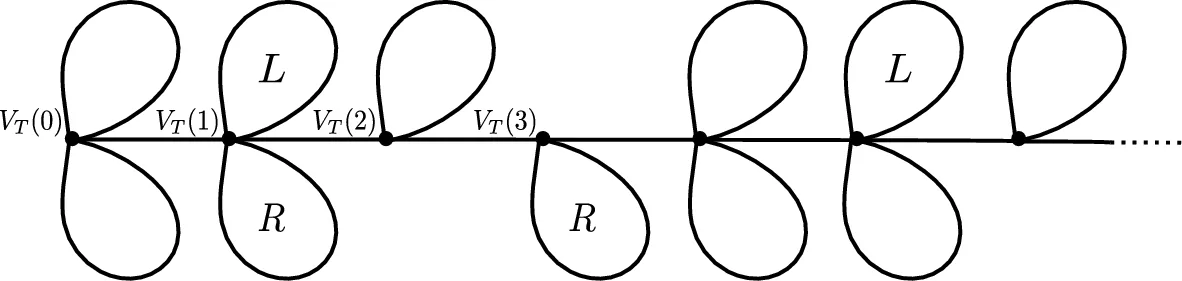
An infinite tree $T\in \hat{\mathcal {T}}^*_\infty$ with its first two RL pairs

## Data Availability

No datasets were generated or analyzed during the current study.

## Local Metrics and Continuity

For completeness, we provide here some details on the constructions of the infinite objects $\textsf{SP}$, $\textsf{N}$, $\textsf{P}$, and $\textsf{S}$.

Recall the sets $\mathcal {T}$ (finite plane trees with no vertices of outdegree 1) and $\mathcal {T}^*$ (trees in $\mathcal {T}$ where the leftmost child of the root is a leaf) from Sect. 2.2. Denote by $\overline{\mathcal {T}}$ the set of (possibly infinite) plane trees, which have no vertices of outdegree 1, and by $\mathcal {T}_\infty = \overline{\mathcal {T}}\setminus \mathcal {T}$ the infinite trees. Denote by $\overline{\mathcal {T}}^*$ the set of trees in $\overline{\mathcal {T}}$ such that the leftmost child of the root is a leaf and let $\mathcal {T}^*_\infty = \overline{\mathcal {T}}^*{\setminus } \mathcal {T}^*$. As for $\mathcal {T}^*$, we take trees in $\overline{\mathcal {T}}^*$ to be labeled so that vertices in even generations have label *P* and vertices in odd generations have label *S*.

We define *local metrics* and *local topologies* on $$\overline{\mathcal {T}}$$, $$\overline{\mathcal {T}}^*$$, and $$\mathcal{S}\mathcal{P}$$ as follows. First, for any graph *G* with a distinguished vertex *v*, we let $$B_r(G;v)$$ denote the subgraph of *G* spanned by vertices at distance ≤r from the vertex *v*. For two graphs *G* and $G^{\prime }$ belonging to some set of graphs $$\mathcal {G}$$, we let

$$\begin{aligned} d_{\mathcal {G}}(G,G')=\frac{1}{1+\max \{r\in \mathbb {N}: B_r(G;v)=B_r(G';v)\}}. \end{aligned}$$

The topology generated by this metric is called the *local topology* with respect to *v*. In the case when $\mathcal {G}$ is one of the sets $\overline{\mathcal {T}}$, $\overline{\mathcal {T}}^*$ we take v=∅ and use the notation $d_\mathcal {T}$ for the metric in both cases. When $\mathcal {G}$ is the set $\mathcal{S}\mathcal{P}$, we use $v=*_0$.

Using the local metric, we can define *infinite* series–parallel maps. Namely, we let $\overline{\mathcal{S}\mathcal{P}}$ denote the completion of $\mathcal{S}\mathcal{P}$ with respect to the metric $d_{\mathcal{S}\mathcal{P}}$. Then, $\mathcal{S}\mathcal{P}_\infty :=\overline{\mathcal{S}\mathcal{P}}\setminus \mathcal{S}\mathcal{P}$ is the set of infinite series–parallel maps, which are formally equivalence classes of Cauchy sequences of finite series–parallel maps. We now show how to extend the bijection $\varphi ^*:\mathcal {T}^*\rightarrow \mathcal{S}\mathcal{P}$ of Sect. 2.3 to infinite trees and maps.

A *spine* of an infinite tree is an infinite path (*V*(0), *V*(1), *V*(2), ...) such that V(0)=∅ and V(i+1) is a child of *V*(*i*) for all i≥0. At every vertex *V*(*i*) on the spine, there is one subtree rooted on the left of the spine and another one on the right of the spine, which we refer to as the left and right *outgrowths* of the spine. Let $\hat{\mathcal {T}}^*_\infty$ be the subset of $\mathcal {T}^*_\infty$ consisting of trees with a *unique* spine and such that this unique spine has infinitely many outgrowths in odd generations on both the left and the right.

Now let $T\in \hat{\mathcal {T}}^*_\infty$ with unique spine $(V_{T}(0),V_{T}(1),V_{T}(2),...)$. We inductively define a sequence $(i_k,j_k)_{k\ge 1}$ of pairs of *odd* integers $i_k\le j_k$ as follows, see Fig. 7 for an illustration. First, $i_1$ is the smallest odd integer *i* so that $V_T(i)$ has a non-empty outgrowth on the right of the spine, and $j_1$ is the smallest odd integer $j\ge i_1$ so that $V_T(j)$ has a non-empty outgrowth on the left of the spine. Inductively, $i_{k+1}$ is the smallest odd integer $i>j_k$ so that $V_T(i)$ has a non-empty outgrowth on the right of the spine, and $j_{k+1}$ is the smallest odd integer $j\ge i_{k+1}$ so that $V_T(j)$ has a non-empty outgrowth on the left of the spine. We call $(V_T(i_k),V_T(j_k))_{k\ge 1}$ the *RL pairs* of *T*. Since $i_k,j_k$ are odd, $V_T(i_k),V_T(j_k)$ are labeled by *S*. Note that $\hat{\mathcal {T}}^*_\infty$ is defined so that *T* has infinitely many RL pairs.

### Proposition A.1

Let $[T]_m:= B_m(T;\varnothing )$ denote the first *m* generations of *T*.

- If $T \in \hat{\mathcal {T}}^*_\infty$, then $\varphi ^*([T]_m)$ is a Cauchy sequence with respect to $d_\mathcal{S}\mathcal{P}$. Thus, $\varphi ^*(T):= \lim _{m\rightarrow \infty } \varphi ^*(\left[ T\right] _{m})$ gives a well-defined function $\mathcal {T}^*\cup \hat{\mathcal {T}}^*_\infty \rightarrow \overline{\mathcal{S}\mathcal{P}}$.
- If a sequence $(T_{n})_{n\ge 1}$ in $\mathcal {T}^*\cup \hat{\mathcal {T}}^*_\infty$ converges to $T\in \mathcal {T}^*\cup \hat{\mathcal {T}}^*_\infty$ with respect to the metric $d_\mathcal {T}$, then $\varphi ^*(T_{n}) \rightarrow \varphi ^*(T)$ with respect to $d_\mathcal{S}\mathcal{P}$.

### Proof

The key observation is that if $T \in \hat{\mathcal {T}}^*_\infty$ and v∈T is a leaf that is beyond the first *r* RL pairs, then for any *m* large enough such that $v\in [T]_m$, the edge $\textbf{e}_v$ corresponding to *v* in $\varphi ^*([T]_m)$ is at least at distance *r* from both poles $*_0$ and $*_1$. This follows from the recursive description of $\varphi ^*$, where the non-empty outgrowths of each RL pair become non-empty networks placed in series on either side between $\textbf{e}_v$ and the poles $*_0$ and $*_1$. Each of these networks contributes to the distance from the poles to $\textbf{e}_v$ at least by one and to the total distance at least by *r*. Due to this observation, the proof can be completed as follows.

- If $[T]_m$ contains the first r+1 RL pairs for all $m >m_{0}(r)$ then $\forall m,m^{\prime }>m_{0}(r)$, $B_r(\varphi ^*([T]_m)=B_r(\varphi ^*([T]_{m'}))$ since all the edges beyond the first r+1 RL pairs do not belong to $B_r(\varphi ^*([T]_m))$.
- Let $(T_{n})_{n\ge 1}$ be a sequence in $\overline{\mathcal {T}}^*$ converging to $T \in \mathcal {T}^*\cup \hat{\mathcal {T}}^*_\infty$. If $T \in \mathcal {T}^*$, then $T_{n}=T$ for every *n* large enough so that $\varphi ^*(T_{n}) \rightarrow \varphi ^*(T)$ is obvious. Now, we assume that $T \in \hat{\mathcal {T}}^*_\infty$. Let *R* be such that $[T]_R$ contains the first r+1 RL pairs. There exists a number $n_{0}(R)$ so that for some $n>n_{0}(R)$, $[T_{n}]_R= [T]_R$. For such *n*, it holds that $B_r(\varphi ^*([T_{n}]_R))=B_r(\varphi ^*([T]_R))$.

□

Since the local limit $\hat{\textsf{T}}$ of the trees $\textsf{T}_n$ almost surely belongs to $\hat{\mathcal {T}}^*_\infty$, it follows that the local limit $\textsf{SP}_\infty =\varphi ^*(\hat{\textsf{T}})$ of $\textsf{SP}_n$ is a well-defined element of $\mathcal{S}\mathcal{P}_\infty$.
